# Atractylodin Induces Myosin Light Chain Phosphorylation and Promotes Gastric Emptying through Ghrelin Receptor

**DOI:** 10.1155/2017/2186798

**Published:** 2017-08-13

**Authors:** Yu Bai, Yan-hua Zhao, Jian-ya Xu, Xi-zhong Yu, Yun-xia Hu, Zhi-qiang Zhao

**Affiliations:** ^1^The First College of Clinical Medicine, Nanjing University of Chinese Medicine, Xianlin Avenue 138, Qixia District, Nanjing 210023, China; ^2^Jiangsu Key Laboratory of Pediatric Respiratory Disease, Institute of Pediatrics, Nanjing University of Chinese Medicine, Xianlin Avenue 138, Qixia District, Nanjing 210023, China; ^3^Medical Research Centre, First College of Clinical Medicine, Nanjing University of Chinese Medicine, Xianlin Avenue 138, Qixia District, Nanjing 210023, China

## Abstract

Atractylodin is one of the main constituents in the rhizomes of* Atractylodes lancea* Thunb., being capable of treating cancer cachexia-anorexia and age-related diseases as an agonist of growth hormone secretagogue receptor (GHSR). GHSR was herein expressed in human gastric smooth muscle cells (HGSMCs) and activated by ghrelin receptor agonist L-692,585. Like L-692,585, atractylodin also increased Ca^2+^ and enhanced the phosphorylation of myosin light chain (MLC) through GHSR in HGSMCs. In addition, atractylodin promoted gastric emptying and MLC phosphorylation in the gastric antrum of mice also through GHSR. Collectively, atractylodin can activate GHSR in gastric smooth muscle, as a potential target in clinical practice.

## 1. Introduction

**Figure 1:**
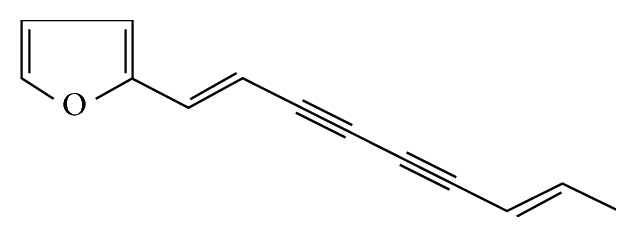
Structure of atractylodin.

*Atractylodes lancea* Thunb. (Asteraceae) has commonly been used to improve gastric function, to stimulate appetite, and to treat gastric diseases in traditional Chinese medicine and East Asia [1], but the underlying mechanism is still unclear. Atractylodin is one of the main constituents in the rhizomes of* A. lancea* (Figure 1), but its pharmacological activities have seldom been studied hitherto. Fujitsuka et al. reported that atractylodin activated growth hormone secretagogue receptor (GHSR) and enhanced ghrelin-induced Ca^2+^ fluorescence in GHSR-expressing cells [2]. Besides, atractylodin has been used to treat tumor-bearing rats and aged mice, being related to GHSR [3].

As an endogenous ligand of GHSR, ghrelin was first isolated from the stomach of rats [4–6], and GHSR mRNA was expressed in the whole gastrointestinal tract and hypothalamus. Besides promoting gastric phase III-like contractions and stimulating growth hormone release, ghrelin also regulates energy balance and stimulates food intake in humans [7] and rodents [8] both centrally and peripherally. Myosin light chain (MLC) plays a pivotal role in regulating muscle contraction in gastric, vascular, and uterine smooth muscles [9, 10]. Phosphorylation of Ser19 in MLC has been highlighted in studies on the regulation of smooth muscle contractile activity. This phosphorylation can be mediated by MLCK, predominantly depending on calmodulin and the concentration of free calcium ions (Ca^2+^) [11].

Probably, GHSR was linked to MLC phosphorylation. As a GHSR agonist, atractylodin may be able to activate GHSR in the gastrointestinal tract, especially the stomach, and to affect gastrointestinal motility. Motivated by this, we herein evaluated the effects of atractylodin on gastric smooth muscle and gastrointestinal motility in vivo and in vitro, aiming to explore its clinical potential.

## 2. Material and Methods

### 2.1. Chemical Compound

Atractylodin with over 98% purity was purchased from Chengdu Pufeide Biological Technology Co., Ltd. (batch number 150112, China). Atractylodin was diluted in 250 mM dimethyl sulfoxide (DMSO) for in vitro test and dissolved in solution containing 1% Tween-80 for in vivo test as previously described [2].

### 2.2. Reagents

Fluorescence probe of Ca^2+^ (Fluo-4 AM) was purchased from Dojindo (CAS: 273221-67-3, Kumamoto, Japan). DMSO (CAS: 67-68-5) and thapsigargin (CAS: 67526-95-8) were from Sigma-Aldrich (St. Louis, MO, USA). GHSR antagonist (D-Lys3)-GHRP-6 was from Bachem (CAS: 136054-22-3, CA, California, USA). Antibodies against phosphorylated and total forms of MLC (#3672 and #3675), RIPA lysis buffer (10x), and phenylmethanesulfonyl fluoride (PMSF, #8553) were from Cell Signaling Technology (Beverly, MA, USA). Phosphatase inhibitors cocktail was from Roche Molecular Biochemicals (CAS: 4906837001, Nutley, NJ, USA). Poly-L-lysine was from ScienCell Research Laboratories (San Diego, CA, USA). Poly-L-lysine stock solution was from ScienCell Research Laboratories (CAS: 0413, Carlsbad, CA, USA). Anti-ghrelin receptor antibody (ab95250) and secondary goat anti-rabbit IgG (H+L) antibody (ab96899) were from Abcam (San Francisco, CA, USA). Ghrelin receptor agonist L-692,585 was from Santa Cruz Biotechnology (CAS: 145455-35-2, Dallas, TX, USA).

### 2.3. Cell Culture

Human gastric smooth muscle cells (HGSMCs, Catalog #2810), smooth muscle cell medium (SMCM, Catalog #1101), penicillin/streptomycin solution (P/S, Catalog number 0503), fetal bovine serum (FBS, Catalog number 0010), and smooth muscle cell growth supplement (SMCGS, Catalog number 1152) were obtained from ScienCell Research Laboratories. A poly-L-lysine-coated culture vessel was prepared in a 37°C incubator overnight (or for a minimum of one hour) and rinsed twice with sterile water before use. HGSMCs were cultured in SMCM which contained 2% (v/v) FBS, 1% (v/v) SMCGS, and 1% (v/v) P/S. Confluent cells were serum-starved for 6 h in basal medium before treatment. Antagonists and other intervening measures were added to cells 30 min before stimuli.

### 2.4. Measurement of Intracellular Ca^2+^ Levels

Intracellular Ca^2+^ level changes were measured by a calcium-specific fluorescent dye described previously [12]. Fluo-4 AM was prepared into a 1 mM stock solution by DMSO before being used at −20°C, and atractylodin was diluted in HBSS (140 mg/L CaCl_2_, 100 mg/L MgCl_2_-6H_2_O, 100 mg/L MgSO_4_-7H_2_O, 400 mg/L KCl, 60 mg/L KH_2_PO_4_, 350 mg/L NaHCO_3_, 8000 mg/L NaCl, 48 mg/L Na_2_HPO_4_, and 1000 mg/L D-glucose) or D-HBSS (100 mg/L MgCl_2_-6H_2_O, 100 mg/L MgSO_4_-7H_2_O, 400 mg/L KCl, 60 mg/L KH_2_PO_4_, 350 mg/L NaHCO_3_, 8000 mg/L NaCl, 48 mg/L Na_2_HPO_4_, and 1000 mg/L D-glucose) to stimulate HGSMCs. The cells were loaded finally with 2 *μ*M Fluoro-4 AM for 30 min at 37°C in PBS. After incubation, the cells were washed mildly three times by HBSS or D-HBSS to eliminate excess Fluo-4 AM. With a confocal microscope (LEICA SP5), the fluorescence of Fluo-4 AM was excited at 488 nm, emitted at 543 nm, and recorded as time-series mode per 3 s. Fluorescent images were analyzed by LEICA SP5 confocal microscope. To survey the representative changes of intensity, the whole screen was measured. In all cases, the changed fluorescence was expressed as the relative intensity compared with the initial value.

### 2.5. Western Blot Analysis of MLC and* p*-MLC

Cells or gastric antrum samples were lysed using an ice-cold lysis buffer (RIPA×1 containing 1% PMSF and 1% phosphatase inhibitors cocktail). Equal amounts of protein per sample (50 *μ*g) were separated by 12% SDS–PAGE and subsequently transferred to polyvinylidene fluoride membranes. After blocking with 3% bovine serum albumin (BSA), the membranes were incubated with purified rat anti-MLC (1 : 2000) and purified mouse anti-phospho Ser19 MLC (1 : 2000) at 4°C overnight. The membranes were then washed and further incubated with different secondary antibodies (1 : 10000), and the bands were visualized by adding enhanced chemiluminescence reagents. The results of Western blot were analyzed by Image J.

### 2.6. Ghrelin Receptor Immunofluorescence Staining

After fixing with 4% paraformaldehyde for 30 min, HGSMCs on coverslips were permeabilized in PBS with 0.2% Triton X-100 for 5 min and incubated with 3% BSA in PBS for the immunocytochemical analysis of ghrelin receptor. The cells were incubated with anti-ghrelin receptor antibody (1 : 200) overnight at 4°C. After extensive washing with PBS, the cells were exposed to DAPI-conjugated secondary antibody for 1 h [13]. The fluorescence images were observed by a laser scanning confocal microscope.

### 2.7. Animals and Gastric Emptying Measurement

Gastric emptying was examined according to a previously described method [14]. Kunming mice were fasted for 12 h before experiment. Atractylodin (0.1, 1, and 10 mg/kg) [2, 3], mosapride citrate (ME) tablet (10 mg/kg) [14] as a positive control, or 0.5% carboxyl methyl cellulose (CMC) (10 mL/kg) as a vehicle control was administered orally (intragastrically) in a volume of 0.1 mL/10 g body weight. Thirty minutes later, a liquid meal containing phenol red (50 mg) suspended in CMC (100 mL) was also administered orally in a volume of 0.3 mL. After 20 min, the animals were sacrificed and the amount of phenol red retained in the stomach was measured. In each experiment, another 4 mice as control were also killed immediately after administration of the liquid meal at zero time (the absorbance of phenol red was used as standard). After decapitation, stomachs were clamped at the pylorus and cardia and then removed. The stomach was cut into several pieces and homogenized in 25 mL of 0.1 M NaOH. The suspension was settled for 60 min at room temperature, and 4 mL of supernatant was added to 0.5 mL of trichloroacetic acid (33% w/v). After centrifugation at 3000 rpm for 10 min, the supernatant was added to 1 mL of 2 M NaOH, and absorbance of the sample was determined at 560 nm with a Type 1800 spectrophotometer. Gastric emptying was calculated according to the following formula: gastric emptying% = 1 − amount of phenol red recovered from test stomach/average amount of phenol red recovered from standard stomachs × 100%. All experiments were performed in accordance with the guidelines for the care and use of laboratory animals and related ethical regulations of Nanjing University of Chinese Medicine.

### 2.8. Calculations and Statistics

All values were expressed as mean ± standard error of mean (SEM). Data were analyzed by one-way analysis of variance, and differences among means were analyzed using Dunnett's test or Fisher's protected LSD multiple comparison test. Tests were performed using SPSS 19.0 system (Chicago, IL, USA).* P* value less than or equal to 0.05 was considered statistically significant.

## 3. Results

### 3.1. Functional GHSR Was Expressed on HGSMCs Surface

**Figure 2:**
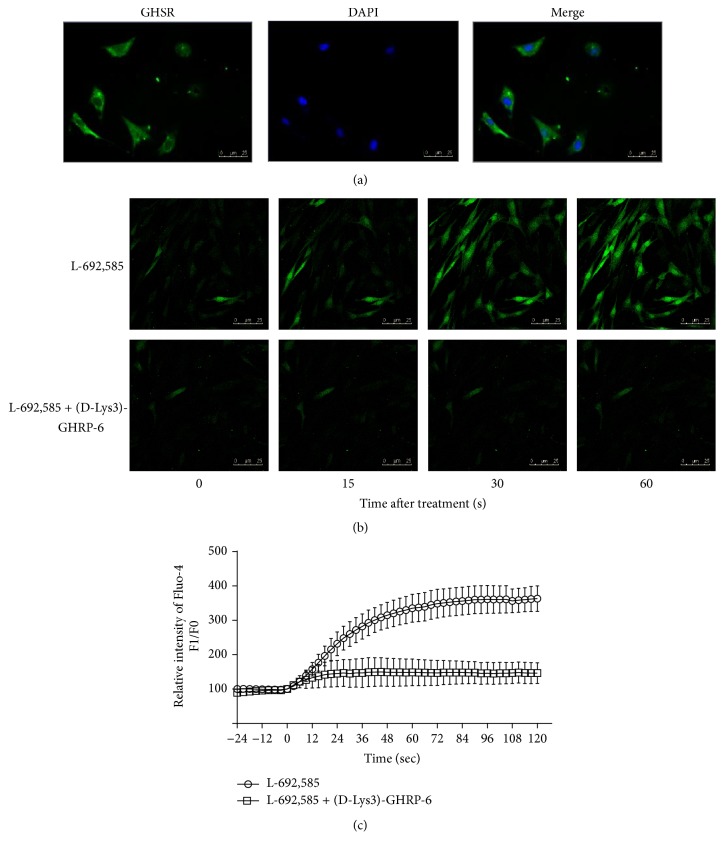
Functional GHSR was expressed on HGSMCs surface. (a) HGSMCs were labeled for GHSR (green). ((b) and (c)) GHSR agonist L-692,585 (10 *μ*M) markedly increased intracellular Ca^2+^ levels in HGSMCs, which was inhibited by GHSR antagonist (D-Lys3)-GHRP-6 (100 *μ*M).

Although GHSR exists in smooth muscle of the whole gastrointestinal tract, it is still necessary to confirm the expression of functional GHSR on the HGSMCs surface. GHSR was markedly expressed on the surface of HGSMCs (Figure 2(a)) and ghrelin receptor agonist L-692,585 evidently increased intracellular Ca^2+^ levels (Figures 2(b) and 2(c)). To further verify whether the response was indeed mediated by GHSR, (D-Lys3)-GHRP-6, a specific GHSR antagonist, was used to pretreat HGSMCs before L-692,585 treatment. As shown in Figures 2(b) and 2(c), after pretreatment with (D-Lys3)-GHRP-6, L-692,585 cannot increase intracellular Ca^2+^ levels in HGSMCs anymore. Taken together, functional GHSR was expressed on the surface of HGSMCs.

### 3.2. Atractylodin Increased Ca^2+^ in HGSMCs through GHSR

**Figure 3:**
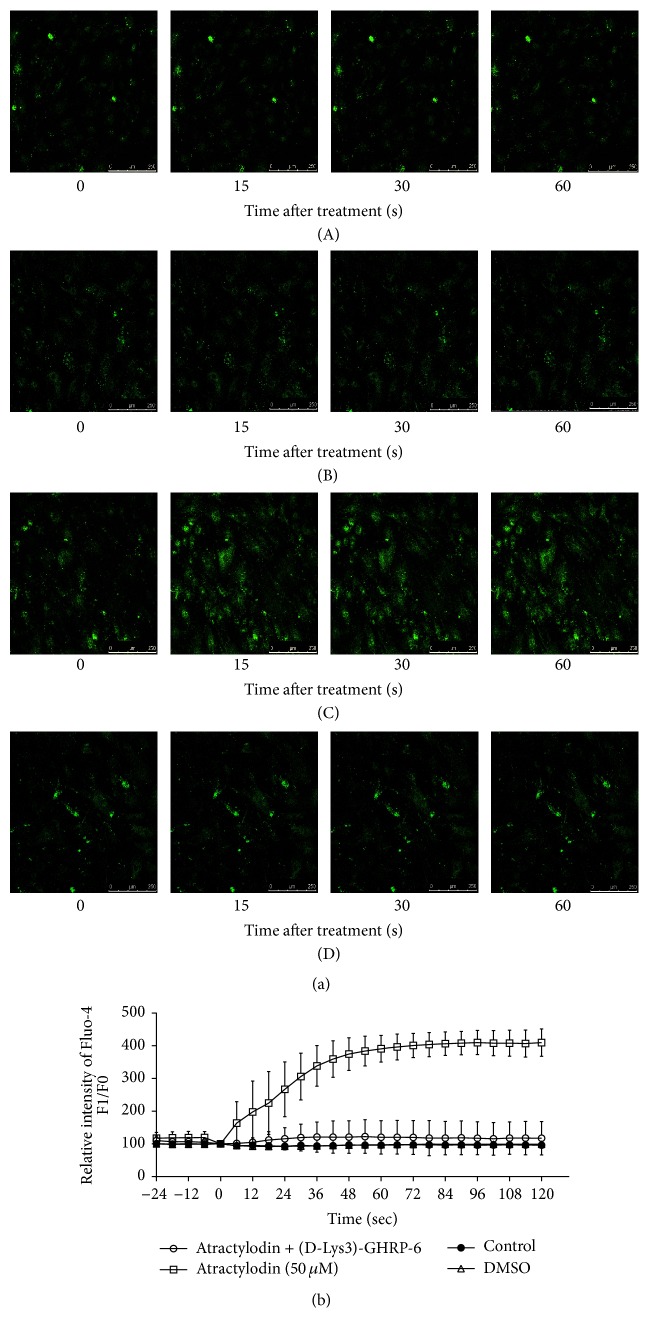
Atractylodin increased Ca^2+^ in HGSMCs. (a) HGSMCs were incubated with Fluo-4 AM (2 *μ*M) for 30 min and then treated with vehicle (A), DMSO (B), and 50 *μ*M atractylodin (C) or pretreated with (D-Lys3)-GHRP-6 (D). (b) In all cases, the changed fluorescence was expressed as the relative intensity compared with the initial value (*n* = 3). Values were expressed as mean ± SEM.

Fluo-4 AM, a fluorescent Ca^2+^-sensitive dye, was used to measure atractylodin-induced Ca^2+^ release in HGSMCs. Atractylodin rapidly elevated intracellular Ca^2+^ level, which was not related to DMSO or vehicle (Figures 3(a)(A), 3(a)(B), 3(a)(C), and 3(b)) in HBSS. Furthermore, to verify whether increased intracellular Ca^2+^ level was related to GHSR, we used (D-Lys3)-GHRP-6 to block the activity of GHSR. After treatment with (D-Lys3)-GHRP-6, atractylodin could not significantly increase the green fluorescence of Ca^2+^ in HGSMCs (Figures 3(a)(C) and 3(b)). Taken together, atractylodin increased Ca^2+^ in HGSMCs through GHSR.

### 3.3. Atractylodin Induced MLC Phosphorylation through GHSR and Ca^2+^ in HGSMCs

**Figure 4:**
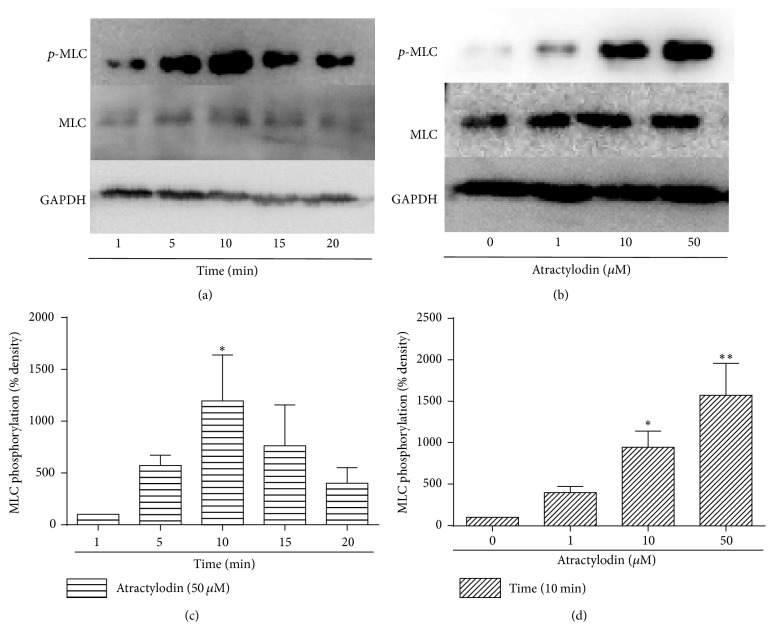
Atractylodin induced MLC phosphorylation in HGSMCs. (a, b) HGSMCs were treated with 50 *μ*M atractylodin for different times (1, 5, 10, 15, and 20 min). ^*∗*^*P* < 0.01 versus 1 min group. (c, d) Then HGSMCs were treated with different doses of atractylodin (0, 1, 10, and 50 *μ*M) for 10 min (*n* = 3 or 4). ^*∗*^*P* < 0.05 and ^*∗∗*^*P* < 0.01 versus 0 *μ*M group. Values were expressed as mean ± SEM.

**Figure 5:**
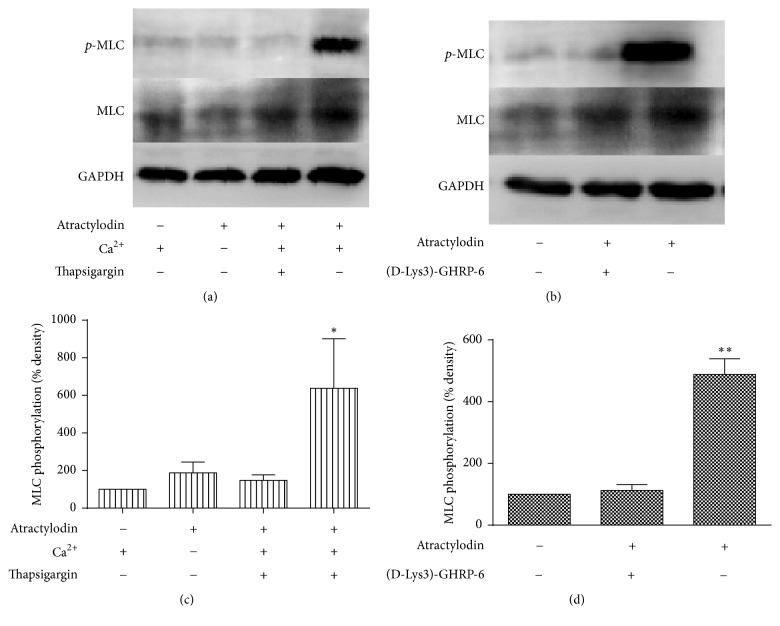
Atractylodin induced MLC phosphorylation in HGSMCs through intracellular and extracellular Ca^2+^ and GHSR. (a, b) HGSMCs were pretreated with thapsigargin (2.5 *μ*M) or (D-Lys3)-GHRP-6 (100 *μ*M) for 30 min and washed three times and incubated with HBSS or D-HBSS to exclude Ca^2+^ interference. Then all groups were treated with 50 *μ*M atractylodin for 10 min (*n* = 4). ^*∗*^*P* < 0.05 versus other groups. ^*∗∗*^*P* < 0.01 versus other groups. Values were expressed as mean ± SEM.

We then studied whether atractylodin induced MLC phosphorylation in HGSMCs. Atractylodin at 50 *μ*M significantly induced MLC phosphorylation in 10 min (Figures 4(a) and 4(b)). The effect was also obviously dose-dependent (Figures 4(c) and 4(d)). To further research the relationship between MLC phosphorylation and Ca^2+^, Ca^2+^-free solution (D-HBSS) and thapsigargin were used to abolish the extracellular and intracellular effects of Ca^2+^ as previously described [15]. D-HBSS and thapsigargin significantly inhibited atractylodin-induced MLC phosphorylation (Figures 5(a) and 5(b)), and (D-Lys3)-GHRP-6 functioned identically (Figures 5(c) and 5(d)). Collectively, Ca^2+^ and GHSR may be both involved in the mechanism by which atractylodin induced MLC phosphorylation.

### 3.4. Atractylodin Promoted Gastric Emptying and MLC Phosphorylation through GHSR In Vivo

**Figure 6:**
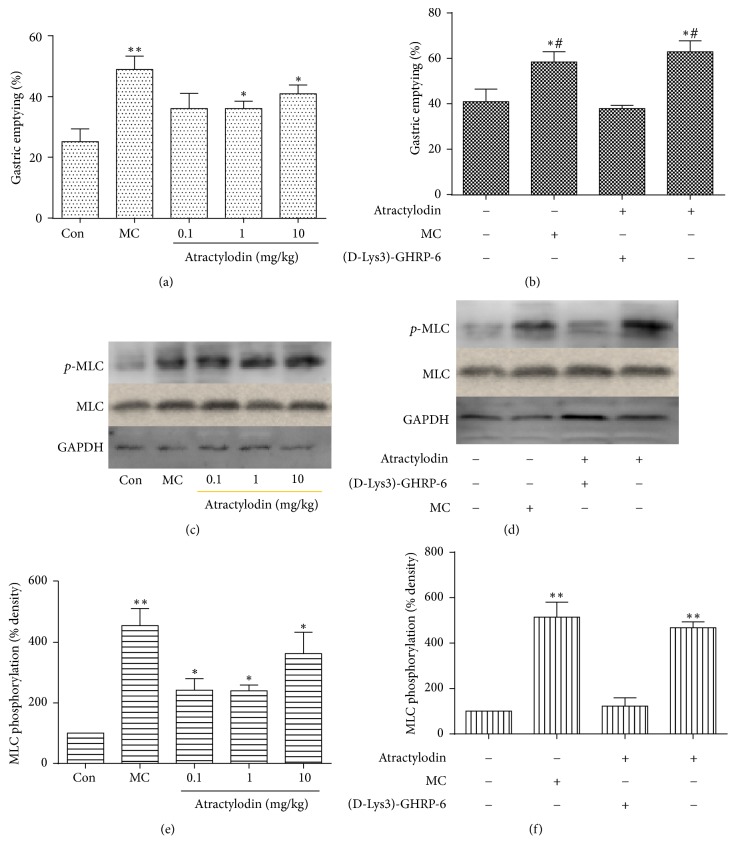
Atractylodin promoted gastric emptying and MLC phosphorylation. (a, b) Mice were sacrificed to measure the gastric emptying rate after treatment with different doses of atractylodin (0.1, 1, and 10 mg/kg) and ME (10 mg/kg). (D-Lys3)-GHRP-6 (10 mg/kg) was continuously injected 3 days or 30 min before atractylodin treatment (*n* = 7 or 8). *∗* or *∗∗* means* P* < 0.05 or 0.01 versus control; # means* P* < 0.05 versus (D-Lys3)-GHRP-6 groups. (c, d, e, f) MLC phosphorylation in the gastric antrum of mice (*n* = 3 or 4). *∗* or *∗∗* means* P* < 0.05 or 0.01 versus control; # means* P* < 0.05 versus (D-Lys3)-GHRP-6 groups. Values were expressed as mean ± SEM.

Based on the results above, atractylodin was related to GHSR in vitro. Given that ghrelin can promote gastric phase III-like contractions through GHSR [5, 16], atractylodin may also be related to GHSR in vivo. Therefore, we first designed an experiment to evaluate the gastric emptying effect of atractylodin. Forty Kunming mice were divided into five groups. Compared with the control group, 10 mg/kg atractylodin and ME significantly increased the gastric emptying rate (Figure 6(a)) and also induced MLC phosphorylation in the gastric antrum (Figures 6(c) and 6(e)). Then (D-Lys3)-GHRP-6 was intraperitoneally injected 30 min before atractylodin treatment to study whether such increased rate was associated with GHSR. Compared with the control group, atractylodin did not increase the gastric emptying rate after (D-Lys3)-GHRP-6 injection (Figure 6(b)) or induce MLC phosphorylation in the gastric antrum (Figures 6(d) and 6(f)).

## 4. Discussion

Ghrelin and GHSR have been used as the targets for treating many diseases such as cancer anorexia-cachexia, functional gastrointestinal disorders, and malnutrition, even prolonging the survival of patients with age-related syndromes [2, 3, 17, 18]. However, only one GHSR agonist, anamorelin, has been approved for phase III clinical trials [19, 20].

Many herbs have been used in traditional Chinese medicine to treat gastrointestinal diseases, following unclear mechanisms. Probably, some of them contain chemical components that can activate the ghrelin signaling pathway through GHSR. In traditional Chinese medicine, the rhizomes of* A. lancea* have commonly been used to regulate gastrointestinal function and to relieve anorectic symptom. Therefore, the rhizomes may contain components that can activate the ghrelin signaling pathway. Extracted from the rhizomes of* A. lancea*, atractylodin has recently been related to GHSR by Japanese researchers [2, 3]. They found atractylodin enhanced the ghrelin-induced Ca^2+^ fluorescence in GHSR-expressing cells, prolonged the survival of tumor-bearing rats, and alleviated age-related diseases in klotho-deficient, SAMP8, and aged ICR mice.

As a specific receptor of ghrelin, GHSR has been found in smooth muscle of the whole gastrointestinal tract, including gastric smooth muscle [21]. To demonstrate our hypothesis that atractylodin also affected gastric smooth muscle through GHSR, we first tested whether it intervened with the intracellular Ca^2+^ level of HGSMCs. The fluorescence of Ca^2+^, which was enhanced after atractylodin treatment in a few seconds (Figure 2(a)), was observably blocked by (D-Lys3)-GHRP-6 (Figure 2(b)), suggesting that atractylodin upregulated the intracellular Ca^2+^ level of HGSMCs through GHSR. Since GHSR and MLC phosphorylation in smooth muscle cells were associated with gastric motility, we then explored whether atractylodin promoted MLC phosphorylation in HGSMCs. Atractylodin significantly induced MLC phosphorylation in HGSMCs dose- and time-dependently (Figure 3(a)(A–D)), which was also inhibited by (D-Lys3)-GHRP-6, so atractylodin indeed induced MLC phosphorylation through GHSR (Figures 4(c) and 4(d)). Additionally, we evaluated the effects of calcium on MLC phosphorylation. Given that intracellular and extracellular Ca^2+^ functions were abolished by Ga^2+^-free solution and thapsigargin, they were involved in the process of MLC phosphorylation (Figures 4(a) and 4(b)). MLC phosphorylation is a key process in smooth muscle contraction and gastric emptying [22], so we studied the effects of atractylodin in vivo. After treatment with atractylodin for 30 min, the gastric emptying rates of the MC group and atractylodin groups at different doses were augmented compared with that of the control group, but only MC and 10 mg/kg atractylodin gave significant differences (Figure 5(a)). Similar to gastric emptying, MLC phosphorylation was also induced by MC as well as 0.1 mg/kg and 10 mg/kg atractylodin (Figures 6(c) and 6(e)). Furthermore, compared with the control group, atractylodin failed to increase the gastric emptying rate after (D-Lys3)-GHRP-6 injection (Figure 5(b)) or induce MLC phosphorylation in the gastric antrum (Figures 6(d) and 6(f)). In short, atractylodin promoted gastric emptying and induced MLC phosphorylation through GHSR both in vivo and in vitro.

It is well-documented that tumor-bearing rats and aged mice can be treated with atractylodin by activating GHSR. In combination with the results herein, atractylodin may also be applicable to the treatment of functional dyspepsia typified by gastric emptying delay. Actually, atractylodin is of great clinical potential, because GHSR is expressed in the gastrointestinal tract, hypothalamic NYP/AgRP nerve [22], human monocytes and T cells [23], pancreatic, lymphoid, and reproductive tissues [24], and even some cancer cell lines [25, 26]. Atractylodin can inhibit interleukin-6 expression in human mast cells [6], mitigate jejunal inflammation, and increase or decrease* p*-MLC in the jejuna of diarrhea- or constipation-predominant rats [27], maybe involving GHSR. Nevertheless, whether GHSR plays the same role in all organs remains elusive, which needs in-depth studies.

## 5. Conclusion

In conclusion, atractylodin increased Ca^2+^ and enhanced MLC phosphorylation in HGSMCs through GHSR. Moreover, atractylodin facilitated gastric emptying and induced MLC phosphorylation in the gastric antrum of mice, also being related to GHSR. Thus, atractylodin is a potentially eligible drug for treating diseases characterized by gastric emptying delay, such as functional dyspepsia.
